# Imagine to automatize: automatization of stimulus–response coupling after action imagery practice in implicit sequence learning

**DOI:** 10.1007/s00426-023-01797-w

**Published:** 2023-03-04

**Authors:** Stephan F. Dahm, Henri Hyna, Daniel Krause

**Affiliations:** 1 Department of Psychology, Universität Innsbruck, Innsbruck, Austria; 2 UMIT Tirol-Private University for Health Sciences and Health Technology, Hall in Tyrol, Austria; 3 Department of Exercise and Health, Paderborn University, Paderborn, Germany

## Abstract

Action imagery practice (AIP) describes the repetitive imagination of an action to improve subsequent action execution. Because AIP and action execution practice (AEP) draw on partly similar motor mechanisms, it was assumed that AIP may lead to motor automatization, which is observable in a reduction of dual-task costs after AEP. To investigate automatization in AIP, we compared dual-task and single-task performance in practice and random sequences in pretests and posttests. All participants practiced serial reactions to visual stimuli in ten single-task practice sessions. An AIP group imagined the reactions. An AEP group and a control practice group executed the reactions. Practice followed a sequential sequence in AIP and AEP but was random in control practice. In dual-task test conditions, tones were counted that appeared in addition to the visual stimuli. RTs decreased from pretest to posttest in both practice and random sequences in all groups indicating general sequence-unspecific learning. Further, RTs decreased to a greater extent in the practice sequence than in the random sequence after AIP and AEP, indicating sequence-specific learning. Dual-task costs—the difference between RTs after tone and no tone events—were reduced independent from the performed sequence in all groups indicating sequence-unspecific automatization. It is concluded that the stimulus–response coupling can be automatized by both, AEP and AIP.

**Supplementary Information:**

The online version contains supplementary material available at 10.1007/s00426-023-01797-w.

## Introduction

Action imagery practice (AIP, also called motor imagery practice or mental practice) designates the repeated use of action imagery with the idea to improve motor performance (Jeannerod, 1995). AIP serves as an alternative form of practice when action execution practice (AEP) is not possible due to injury or because the setting or material is unavailable. Moreover, AIP serves as an amendment to AEP because learning may involve different action representation types (Dahm et al., 2022).

Compared to control groups, AIP has been shown to facilitate subsequent action execution, however to a lesser degree than AEP (Driskell et al., 1994; Simonsmeier et al., 2021; Toth et al., 2020). Improvements after AIP have already been shown on different dimensions of learning: reaction time (RT: Kraeutner et al., 2016; Sobierajewicz et al., 2016), spatial accuracy in goal-directed ballistic tasks (Taylor & Shaw, 2002; Weber & Doppelmayr, 2016), visuomotor adaptation (Michel et al., 2013), and sequence learning (Dahm & Rieger, 2023; Dahm et al., 2022; Land et al., 2016). Further, improvements after AIP have been shown on different levels of action complexity: unimanual coordination (Michel et al., 2013), bimanual coordination (e.g., White & Hardy, 1995), and whole body coordination (e.g., Wakefield & Smith, 2009). Further, AIP has been shown to facilitate other areas of motor performance like stretching and flexibility (Guillot et al., 2010b), and muscle strength (Reiser et al., 2011; Scott et al., 2018). Besides these findings showing a broad effectiveness of AIP, there is no research on how AIP affects the need for attentional control in motor learning (i.e., motor automaticity). Knowledge about the effects of AIP on motor automaticity and its practice-induced change (i.e., motor automatization) is crucial to optimize the goal-directed use of AIP in different stages of motor learning. Therefore, the present study aims to shed light on automatization after AIP.

In AEP, most models of skill acquisition are based on different learning stages and postulate a decrease of demand for attentional control in the course of learning (Chein & Schneider, 2012; Fitts & Posner, 1967). With regard to motor sequence learning guided by external stimuli, it is expected that sequence execution is controlled by single reactions to the external stimuli before learning sets in (i.e., reaction mode; Verwey et al., 2015). Then within the first learning stages, extrinsic visual–spatial representations of the sequence guide the sequence execution (i.e., associative mode) while still heavily demanding for cognitive resources. The learner relies on working-memory dependent processes where spatially coded, effector-independent sequence information (i.e., environment-based representations) is used for sequence execution (Hikosaka et al., 2002; Verwey et al., 2015). In these early stages of learning, spatially represented motor commands are generated and translated to a motor code in a step-by-step manner relying on neural substrates in the prefrontal cortex (spatial representation) and premotor cortex (translation).

With further practice, attention-dependent visual–spatial representations (of the fast-learning system) become redundant as the motor system with its effector-dependent motor representations (of the slow learning system) takes over sequence production. By this, the need for attention during sequence production is reduced. The resulting motor automaticity assumingly occurs due to chunking processes that bind sequence elements which reduces attentional demands in motor control (chunking mode; Immink et al., 2020; Verwey, 1996, 1999; Verwey et al., 2015). These practice-related changes are further accompanied by a shift in neural substrates from frontal to parietal areas being activated during sequence production (Lohse et al., 2014; Sakai et al., 1998). In later stages of learning, motor representations can be accessed with minimal reliance on working-memory-dependent resources (Chein & Schneider, 2012) provoking motor automaticity as indicated by decreased dual-task costs (Abernethy et al., 2012; Fitts & Posner, 1967).

Effects of AIP are often linked to the mere cognitive aspects of action representations, e.g., learning of sequences in a spatial code (Driskell et al., 1994; Jeannerod, 1995), but there is also evidence that motor tasks with less obvious cognitive demands are facilitated by AIP (for meta-analyses: Driskell et al., 1994; Toth et al., 2020). It has been shown that motor learning could not be explained by perceptual learning when it occurred after AIP in the absence of sensory feedback (Ingram et al., 2019). Even strength gains can be achieved with AIP (e.g., Reiser et al., 2011). Whether AIP contributes to the development of representations in a motor code inducing motor automatization is still a research desideratum. Several findings support the idea that motor representations indeed can be acquired with AIP (Ingram et al., 2019; Land et al., 2016; Michel et al., 2013; Reiser et al., 2011). Electromyographic measures in AIP showed mixed findings of the presence or absence of subliminal muscle activations without actual kinematic effects as an indicator that AIP indeed evokes motor programming from the brain toward the muscles (Guillot et al., 2010a). The presence of qualitatively similar but quantitatively reduced muscle activation patterns as compared to AEP seems to be more likely, when a first-person perspective is taken in AIP (Harris & Robinson, 1986). Moreover, brain mapping studies have shown an overlap of involved neural substrates in action execution and action imagery in areas that contribute to (automatic) motor control in later stages of learning such as the primary motor cortex or the basal ganglia (Ladda et al., 2021; Lorey et al., 2013; Lotze et al., 1999; Munzert et al., 2009). Therefore, we expected that AIP does contribute to the acquisition of motor representations that lead to automatization effects. Such automatization effects are usually reflected in the reduction of dual-task costs indicating less dual-task interference (Abernethy et al., 2012; Koch et al., 2018).

In AEP, the degree of automaticity is not solely influenced by the number of repetitions in practice, but also moderated by a number of different practice variables such as focus of attention (Kal et al., 2013), schedule of task difficulty (Maxwell et al., 2001), and the frequency of augmented feedback (Krause et al., 2018). For instance, automatization was observed when feedback was presented on some trials, but not when feedback was presented on every trial (Krause et al., 2018). In AIP, any kind of action feedback is not available, but needs to be imagined (Dahm & Rieger, 2019a, 2019b; Kilteni et al., 2018). Hence, on the one hand, action execution may be more automatic after AIP than after AEP, as there is a limited possibility of error feedback inducing a higher involvement of attention-dependent processing (Krause et al., 2018). In contrast, focusing explicitly on any kind of feedback during AIP may involve even more attention, thereby reducing automatization effects. Further, imagined feedback in AIP is more positive than actual feedback in AEP (Dahm & Rieger, 2019a). Taking into account that AEP positive feedback drives dopamine-dependent learning (Glimcher, 2011) and motor automatization (Agethen & Krause, 2016; Zobe et al., 2019), this may support automatization in AIP too.

Despite the presence of AIP-induced motor automatization, we expect automatization effects to be smaller in AIP than in AEP using a serial reaction time task (Kraeutner et al., 2016; Solomon et al., 2021), as neural activations of more motor-related areas are less pronounced in action imagery than in action execution (Hardwick et al., 2018; Van der Lubbe et al., 2021), especially in imagery of novel motor tasks (Orlandi et al., 2020). Additionally, subsequent action execution profits less from AIP than from AEP (Simonsmeier et al., 2021; Toth et al., 2020). Further, stronger motor representations have been acquired in AEP than in AIP (Kraeutner et al., 2016, 2020), which has additionally been indicated by contra-lateral transfer (Dahm et al., 2022; Land et al., 2016), while the acquisition of effector-independent visual–spatial representations does not differ between AEP and AIP (Dahm et al., 2022). In intermanual transfer, motor representations in AEP and AIP involved either homologous muscle activation patterns (Land et al., 2016) or information that is solely available in the practiced effector (Dahm et al., 2022). Visual–spatial representations involve extrinsic (environment-based) coordinates that are available in any kind of effectors. We assume that motor representations, but not effector-independent visual–spatial representations, contribute to motor automatization. Therefore, automatization is expected to be stronger in AEP than in AIP when practicing according to an implicit sequence learning paradigm (Dahm & Rieger, 2023; Kraeutner et al., 2016).

## Methods

### Participants

Eighty-two students participated in this online study. Four participants were excluded as they had technical difficulties during the study and, therefore, incomplete datasets. A rigorous outlier analysis was planned up front because participants’ setting could not be controlled for at home, making distractions more likely than in a laboratory. Five participants were excluded because they showed non-commitment in the primary task indicated by error rates above the guessing rate of 25% (M ± SD = 5.1 ± 5.4). Four participants were excluded because they made more than three counting errors during dual-task conditions (M ± SD = 1.2 ± 1.4). Moreover, one participant was excluded as an outlier because reaction time after tones in dual-task conditions in Session 1 was more than three standard deviations above the group mean. The participant most likely disregarded the tone counting task for a period of responses which made it difficult to come back to it when a tone occurred. The remaining 68 participants’ sex, age, education, handedness (laterality index by Oldfield, 1971), and vividness of action imagery (Dahm et al., 2019) are shown in Table 1. All participants gave informed consent. Ethical approval was given by the ethics committee of the university. All participants received course credit for their participation.

**Table 1:** Sociodemographic data of the action imagery practice, action execution practice, and control practice group

	Action imagery practice	Action execution practice	Control practice	*p*
Sex, *N*_female_/*N*_male_	14/10	14/11	8/11	0.530
Age, M ± SD	22.9 ± 3.7	22.2 ± 2.4	22.7 ± 3	0.773
Laterality index, M ± SD	67.5 ± 57.2	60.2 ± 65.8	65.8 ± 68.9	0.915
External visual imagery, M ± SD	1.7 ± 0.6	1.9 ± 0.7	1.9 ± 0.9	0.626
Internal visual imagery, M ± SD	1.8 ± 0.6	1.5 ± 0.6	1.6 ± 0.6	0.268
Kinesthetic imagery, M ± SD	1.7 ± 0.5	1.6 ± 0.5	1.9 ± 0.7	0.252

To compare the practice groups, a *χ*^2^ test was calculated for the distribution of sex and ANOVAs with the factor practice (action imagery, action execution, control action) were computed for the remaining variables

The required sample size was estimated with G*Power (Faul et al., 2007) for an interaction between three groups and four conditions (two cognitive load: single-task vs. dual-task × 2 time: pretest vs. posttest). We assumed a medium effect size of *η*_p_^2^ = 0.06 (*f* = 0.25) and correlations among repeated measures of *r* = 0.3. Alpha was set at 0.05 and the power (1-beta) at 0.8 which resulted in a minimum sample size of *N* = 75 (*n* = 25 per group). Although the estimated sample size was not met in the final sample, the estimated power for 68 participants was 1-beta = 0.76 which is close to the desired power of 0.8.

### Primary task: serial reaction time task

**Fig. 1:**
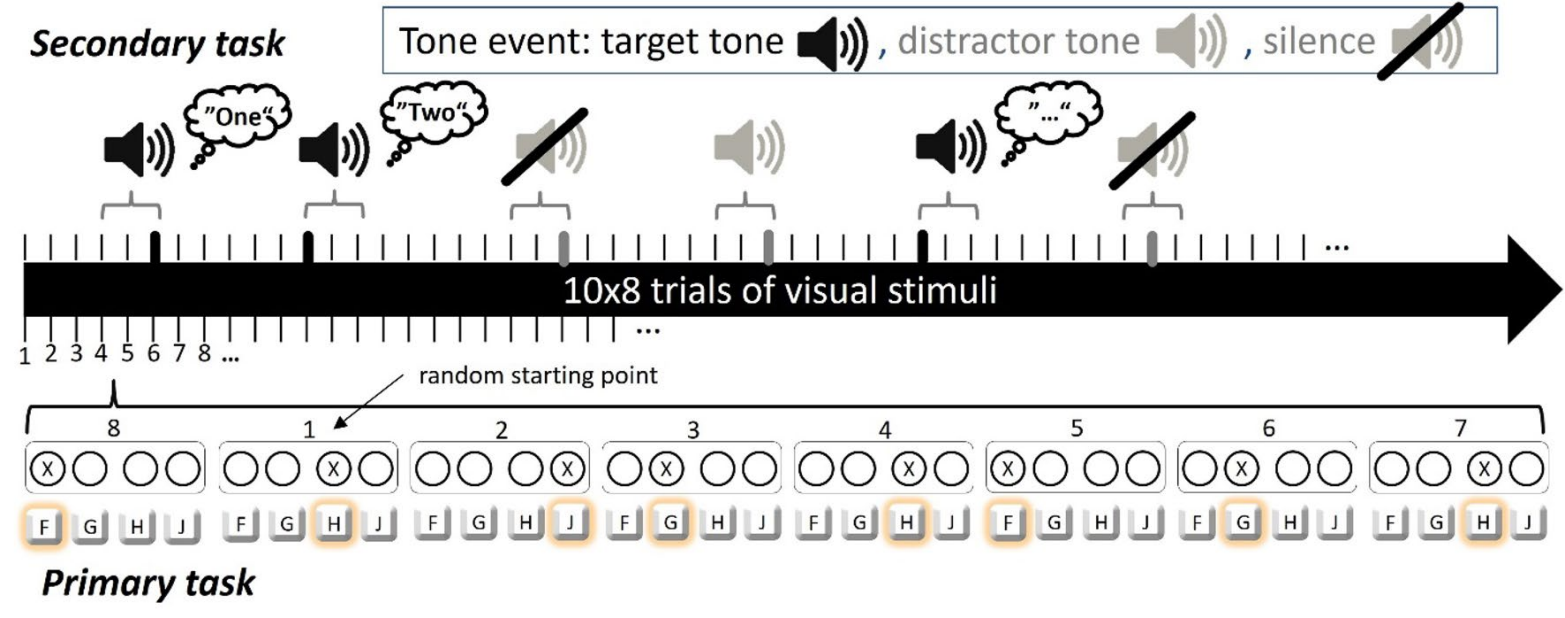
Depiction of the interlaced procedure of primary and secondary task events. Each block included 80 visual stimuli presented either randomly or in the sequence depicted in the example. Within one dual-task block, ten matched tone events (target, distractor, or matched silence) were randomly presented. Participants responded to the visual stimuli with the corresponding target key and counted the target tones

In the serial reaction time task (SRTT), participants react as fast as possible to a series of stimuli (Reber & Squire, 1998). Participants are not informed, that the stimuli (and consequently also their responses) follow a predetermined sequence. In the present study, the responses were performed with the index and middle finger of both hands. The position of the target keys was visual-spatially aligned to the stimuli (see Fig. 1). Visual stimuli showed four circles (*d* = 3.5 cm), two circles left aligned and two circles right-aligned (distance between the centers = 4.5 cm). The distance between the two middle circles was 5.5 cm.

Each block consisted of 80 responses and started with 4 empty circles. After 500 ms, the letter ‘X’ appeared in one of the circles to indicate the corresponding target key. The task was self-paced. Correct and incorrect responses triggered the end of each stimulus, provoking the immediate appearance of the following stimulus. Sequence blocks involved ten repetitions of the eight-element sequence “fhjghfgj”. In each block, one of the eight elements was randomly chosen as starting stimulus. In random blocks, the four targets appeared equally often and without consecutive repetitions. The SRTT was continuous, as there was no pause interval between repetitions of the serial eight-element sequence within the blocks.

### Secondary task: tone counting task

Before every test block that involved the tone counting task (TCT; Verwey et al., 2014), a short tone discrimination task was performed to check and adjust participants’ individual sound setting. The tone discrimination task involved a target tone (440 Hz; scientific pitch notation A4, 100 ms) and a distractor tone (698 Hz; scientific pitch notation: F5; 100 ms) which was first presented together with the instructions. Then one of the tones was presented again (random order). Participants were able to adjust the loudness of the tones to their needs and setting. For this, participants indicated whether the tone was inappropriately quiet or inappropriately loud. In case the volume was appropriate, they indicated whether it is a high tone or a low tone. The discrimination task ended if low and high tones were four times consecutively identified.

In the TCT (Verwey et al., 2014), participants counted the target tones. Each block involved ten tone events. On each tone event, a target tone, a distractor tone, or matched silence event (matched according to positions within the SRTT procedure in relation to target and distractor tones) were randomly presented with the restriction that each event appeared at least twice and maximal six times in a block. In a single-task TCT block, ten TCT events (target tones, distractor tones, matched silence) were randomly presented with a random interval between tone events of 2000–4000 ms. The range of the interval was chosen according to the expected RTs between 250 and 500 ms (Verwey et al., 2014) multiplied by the eight elements of the sequence.

In dual-task blocks, participants were asked to prioritize tone counting to provoke dual-task costs in RTs of the SRTT. In dual-task blocks, each eight-element sequence involved a random presentation of one tone event. The tones appeared simultaneously with the onset of one of the visual stimuli of the SRTT (Verwey et al., 2014). Tone events (target tones, distractor tones, matched silence) were separated by at least five visual stimuli (Fig. 1). At the end of each block, participants reported the number of target tones via mouse click on response boxes on the screen.

### Procedure

The experiment ran on participants’ personal computers using OpenSesame version 3.3.9 (Mathôt et al., 2012). The experimental file including all stimuli and instructions is placed at the Open Science Framework (https://osf.io/nuqx5/?view_only=f95ee1561c7b4b2991633dd7043f7c43). Participation lasted approximately for 15 min in each of the 11 sessions (Table 2). Starting each session, participants were reminded to sit at a table in a quiet room. The time between consecutive sessions was approximately 24 h, but at least one night passed prior to the subsequent session.

**Table 2:** The design of the study includes 11 sessions

Session	1	2–10	11
Experimental phases	Pretest Practice (AIP, AEP, or CP)	Practice (AIP, AEP, or CP)	Posttest (retention, free generation, recognition)

Practice of the serial reaction time task involved either action imagery practice (AIP), action execution practice (AEP), or control practice (CP)

#### Pretest

The first session started with a test phase, which consisted of single-task and dual-task blocks. To avoid order effects of blocks, the order of the blocks was interlaced (ABBA style) as shown in Table 3. After each test block with the TCT, the correct number of targets was presented as feedback (Verwey et al., 2014), while there was no feedback on SRTT performance during test phases. Feedback was only given on TCT performance to strengthen the prioritization of the TCT.

**Table 3:** Pretest and posttest were performed from top to bottom

Task	SRTT	Purpose
TCT		Manipulation check
SRTT	Sequence	Sequence-specific learning
SRTT	Random	Sequence-unspecific learning
SRTT + TCT	Sequence	Sequence-specific dual-task costs
SRTT + TCT	Random	Sequence-unspecific dual-task costs
SRTT + TCT	Random	Sequence-unspecific dual-task costs
SRTT + TCT	Sequence	Sequence-specific dual-task costs
SRTT	Random	Sequence-unspecific learning
SRTT	Sequence	Sequence-specific learning

The test blocks involved either the tone counting task (TCT) as a single-task, the serial reaction time task (SRTT) as a single-task, or a dual-task (TCT + SRTT)

#### Practice phase

The first ten sessions involved a practice phase in which the SRTT was practiced (10 blocks of 80 consecutive responses). Participants were randomly assigned to three groups. In action imagery practice (AIP), participants were asked to imagine pressing the corresponding key with their fingers by focusing on the feeling of the action and seeing the action through their own eyes (first-person imagery). The moment participants imagined pressing the target key, they actually pressed the space key with both thumbs. In action execution practice (AEP) and control practice (CP), they pressed the corresponding keys and simultaneously pressed the space key with both thumbs. In AIP and AEP, participants practiced sequence blocks. In CP, random blocks were practiced. Each practice session lasted approximately for 10 min which is recommended to prevent fatigue during AIP (Driskell et al., 1994; Simonsmeier et al., 2021; Toth et al., 2020). In all groups, participants were not informed about the existence of sequences. After each practice block, feedback about the median response times was given to motivate participants during practice.

#### Posttest

The posttest started according to the same procedure as in the pretest (i.e., measures of retention and dual-task costs). Additionally, participants performed a free generation test. For this, the circles were presented 16 times without the letter ‘X’. Participants were asked to press the target buttons in the order of the practice blocks. This was followed by a recognition test. For this, a sequence block and a random block were performed (16 responses each). After each recognition block, participants rated whether the block coincided with practice (from 1—very unlikely to 9—very likely). The order of the two blocks was randomized.

### Data analysis

RT in the SRTT is the interval between presentation of the visual stimulus and its corresponding response. RTs of the first 16 responses (2 sets of the 8-element sequence) in each block were not taken into analysis assuming that participants needed at least the first sequence set to (implicitly) detect the sequence (Nissen & Bullemer, 1987) and to reduce warm-up effects in general (Steib et al., 2018). Moreover, RTs of an incorrect response and its following response were not taken for analysis. A detailed analysis of error rates in each group and condition is presented in the supplemental material. Of the remaining responses, median RTs were calculated for each block. Then mean RTs were calculated for every two equal blocks (see Table 3). To analyze sequence-unspecific and sequence-specific learning effects, a mixed ANOVA with the between-factor *practice group* (AIP, AEP, CP) and the within-factors time (pretest, posttest), block (sequence, random), and cognitive load (single-task, dual-task) was calculated on RTs.

To analyze automatization effects, we analyzed single RTs within the dual-task blocks. Dual-task costs mainly appeared in one or two RTs shortly after a target or distractor tone (see supplemental material). Therefore, to optimally reflect dual-task costs in the present task, the modal was calculated for the five consecutive RTs following each event (target, distractor, matched silence). Because only events that were followed by correct responses were taken into analysis, two participants of the AEP group showed missing values and were, therefore, excluded from the analysis of dual-task costs.

In addition to RTs, we calculated the number of response triplets in the free generation test that are compatible with the practice sequence and its mirror sequence. This indicates the amount of explicit learning of the sequence structure (Bird & Heyes, 2005). The number of triplets as well as recognition ratings were analyzed with mixed ANOVAs with the between-factor practice group (AIP, AEP, CP) and the within-factor sequence (practice, mirror).

To compare imagination durations and execution durations (Guillot et al., 2012), RTs during practice were analyzed with a mixed ANOVA with the between-factor practice group (AIP, AEP, CP) and the within-factor session (session 1 to session 10). For this analysis, we used responses of the space key which were available in all groups. Additional control analyses on TCT performance and the ratings on strength of representations are presented in the supplemental material.

The following accounts for all ANOVA analyses: If Mauchly’s test indicated that the assumption of sphericity is violated, we report Huynh–Feldt corrected degrees of freedom and *p* values. Further comparisons were conducted using *t* tests with Sidak adjusted pairwise comparisons. Statistical significance was set at *p* < 0.05.

## Results

### Manipulation check: reaction times during practice

**Fig. 2:**
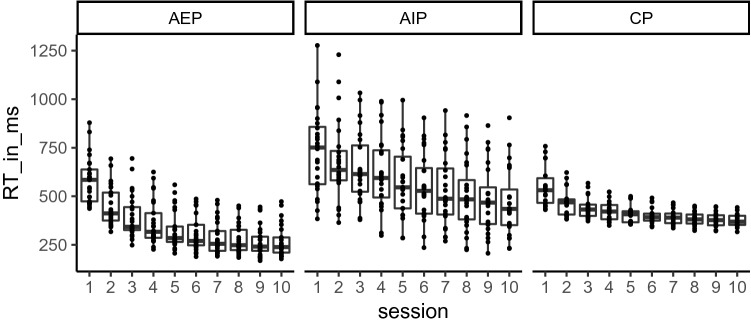
Boxplots of reaction times during practice depending on session (1–10), separately for executed sequence blocks (AEP), imagined sequence blocks (AIP), and executed random blocks (CP)

To evaluate participants’ behavior during practice, we analyzed RTs from the additional key presses on the space bar. The distribution of RTs (in ms) during the ten practice sessions in AEP, AIP, and CP is shown in Fig. 2. An ANOVA with the between-factor practice group (AEP, AIP, and CP) and *session* (1–10) was performed on RTs during practice. The significant main effect *practice group*, *F* (2, 65) = 22.8, *p* < 0.001, *η*^2^_p_ = 0.41, indicated that RTs in the AIP group (*M* = 579 ms) were significantly longer than RTs in the AEP (*M* = 352 ms, *p* < 0.001) and CP group (*M* = 429 ms, *p* < 0.001). The significant main effect *session*, *F* (2.4, 158) = 148.3, *p* < 0.001, *η*^2^_p_ = 0.695, was modified by the significant interaction between *practice group* and *session*, *F* (4.9, 158.5) = 5.6, *p* < 0.001, *η*^2^_p_ = 0.15. In AEP, RTs in Session 10 were significantly shorter than RTs of the first six sessions (*p*_max_ < 0.001). In CP, RTs in Session 10 were significantly shorter than RTs of the first three sessions (*p*_max_ = 0.004). In AIP, RTs in Session 10 were significantly shorter than RTs of the first eight sessions (*p*_max_ < 0.001). Further, RTs in AEP were significantly shorter than in CP from Session 6 onwards (*p*_max_ = 0.029).

### Reaction times in single-task and dual-task blocks

**Fig. 3:**
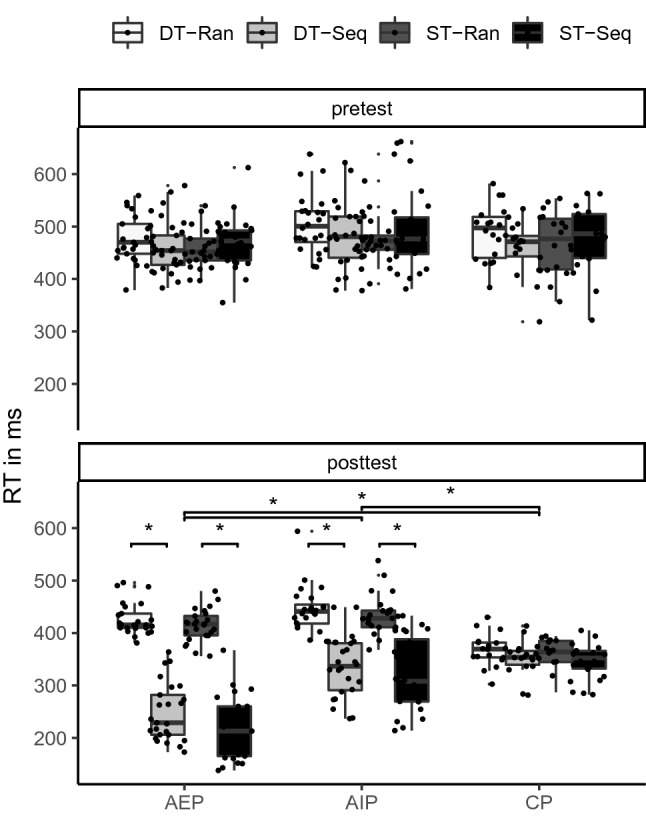
Boxplots of reaction times depending on time (pretest, posttest), sequence (Seq: sequence, Rand: random), and cognitive load (ST: single-task, DT: dual-task), separately for the practice groups (AEP: action execution, AIP: action imagery, CP: control action)

To assess general sequence-unspecific and sequence-specific learning effects, we analyzed RTs in single-task blocks. The distribution of RTs (in ms) of the practice* groups* (AEP, AIP, CP) is shown separately for *time* (pretest, posttest), sequence (sequence, random), and cognitive load (single-task, dual-task) in Fig. 3. Results of the ANOVA are shown in Table 4.

**Table 4:** Statistical values of the ANOVA conducted with the factors practice group (*AEP* action execution, *AIP* action imagery, *CP* control action), time (pretest, posttest), sequence (sequence, random), and cognitive load (single-task, dual-task)

	*F*	df1, df2	*P*	*η*^2^_p_
Practice group	6.0	2, 65	0.004	0.16
Time	713.7	1, 65	< 0.001	0.92
Sequence	285.3	1, 65	< .001	0.81
Cognitive load	77.3	1, 65	< 0.001	0.54
Practice group x time	6.5	2, 65	0.003	0.17
Practice group x sequence	56.9	2, 65	< 0.001	0.64
Practice group x cognitive load	2.0	2, 65	0.151	0.06
Time x sequence	321.8	1, 65	< 0.001	0.83
Time x cognitive load	0.1	1, 65	0.729	< 0.01
Sequence x cognitive load	13.4	1, 65	0.001	0.17
Practice group x time x sequence	91.3	2, 65	< 0.001	0.74
Practice group x time x cognitive load	0.8	2, 65	0.448	0.02
Practice group x sequence x cognitive load	3.9	2, 65	0.025	0.11
Time x sequence x cognitive load	10.3	1, 65	0.002	0.14
Practice group x time x sequence x cognitive load	2.9	2, 65	0.063	0.08

Sequence-unspecific learning was indicated by the significant main effect time*.* RTs became significantly shorter from pretest (*M* = 479 ms) to posttest (*M* = 358 ms).

**Fig. 4:**
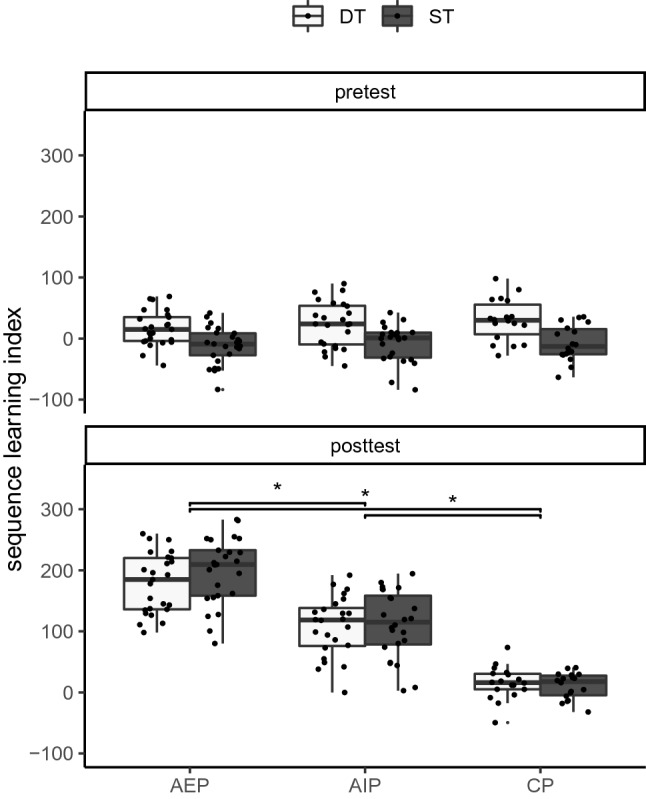
Boxplots of the sequence learning index (Δ RT in random blocks − RT in sequence blocks) depending on time (pretest, posttest), and cognitive load (*ST* single-task, *DT* dual-task), separately for the practice groups (*AEP* action execution, *AIP* action imagery, *CP* control action)

Sequence-specific learning—shorter RTs in sequence than in random blocks—was indicated by the significant main effect sequence, the significant sequence x time interaction, the significant sequence x practice group interaction, and the significant sequence x time x practice group interaction. For a follow-up analysis, the sequence learning index (the difference between RTs in random and sequence blocks) was calculated (see Fig. 4). The analysis revealed that the sequence learning index increased from pretest to posttest in AEP and AIP (*p*_max_ < 0.001), but not in CP (*p* = 0.693). In the pretest, the sequence learning index did not significantly differ between the groups (*p*_min_ = 0.946). In the posttest, the sequence learning index was larger in AEP (Δ = 180 ms) than in AIP (Δ = 104 ms; *p* < 0.001), and smallest in CP (Δ = 11 ms; *p*_max_ < 0.001). Hence, sequence-specific learning occurred in both AEP and AIP, but stronger in AEP than in AIP.

Dual-task costs were indicated by the significant main effect cognitive load, the significant cognitive load x sequence interaction, the significant cognitive load x sequence x practice group interaction, and the significant cognitive load x sequence x time interaction. Although the cognitive load x sequence x practice group x time interaction failed significance (*p* = 0.063), post hoc analyses were performed on this due to the two significant three-way interactions. Further, from a theoretical standpoint, it is essential to take time into account when looking at practice group differences. In random pretest blocks, RTs were significantly longer in dual-task blocks than in single-task blocks (*p*_max_ = 0.002). Similarly in random posttest blocks, RTs were significantly longer in dual-task blocks than in single-task blocks (*p*_max_ = 0.001). In sequence pretest blocks, RTs did not significantly differ between dual-task and single-task blocks in all groups (*p*_min_ = 0.125). In sequence posttest blocks, RTs were significantly longer in dual-task blocks than in single-task blocks after AEP (*p* < 0.001) and after AIP (*p* = 0.002), but not after CP (*p* = 0.671). Hence, dual-task costs were not observed in all conditions in the pretest. Therefore, automatization was analyzed in a more detailed perspective within dual-task blocks in the next step.

### Automatization: comparison of reaction times after tone and matched silence events in the dual-task blocks

**Fig. 5:**
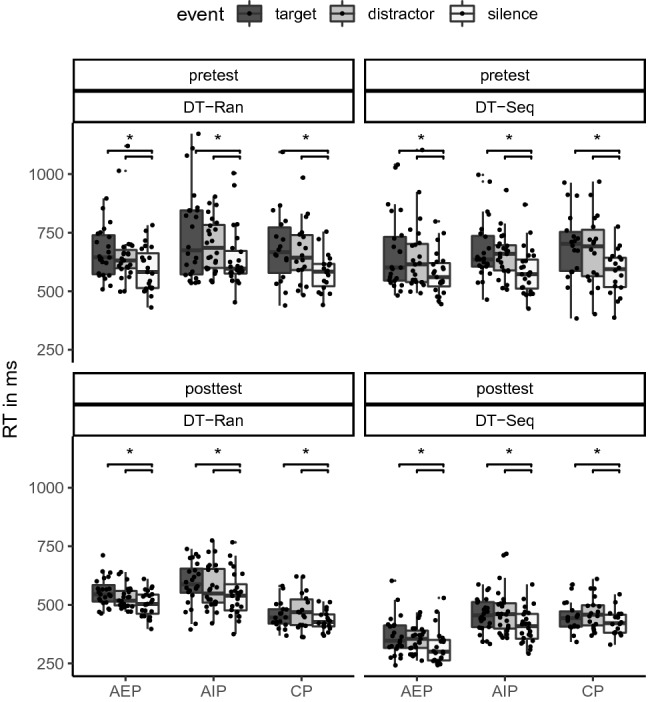
Boxplots of reaction times (RT in ms) shortly after events (target, distractor, silence) depending on time (pretest, posttest) and sequence (sequence, random), separately for the practice groups (*AEP* action execution, *AIP* action imagery, *CP* control action)

To analyze automatization in more detail, a reduced mixed-model ANOVA was performed which focused solely on the factor event (target, distractor, silence) and its’ interactions in the dual-task blocks. The distribution of RTs (in ms) of the practice groups (AIP, AEP, CP) is shown separately for time (pretest, posttest), sequence (sequence, random), and event (target, distractor, silence) in Fig. 5. Two participants had missing values due to errors after events and were therefore excluded from this analysis.

The significant main effect event, *F* (2, 126) = 46.7, *p* < 0.001, *η*^2^_p_ = 0.43, indicated that RTs were significantly longer after target tones (*M* = 586 ms, *p* < 0.001) and distractor tones (*M* = 575 ms, *p* < 0.001) than after matched silence control events (*M* = 520 ms). Most importantly, this indicated dual-task costs in the pretest (in all sequence blocks and practice groups), but also in the posttest. To analyze a possible reduction of dual-task costs, we, therefore, calculated the difference between target and matched silence events in the next step.

From the remaining interactions with event, only the interaction between *event* and *time*, *F* (2, 126) = 10.5, *p* < 0.001, *η*^2^_p_ = 0.14, revealed significance. The interaction of event x time x practice group, *F* (4, 126) = 1.2, *p* = 0.308, *η*^2^_p_ = 0.04, and all other interactions were not significant, *F* < 1.

**Fig. 6:**
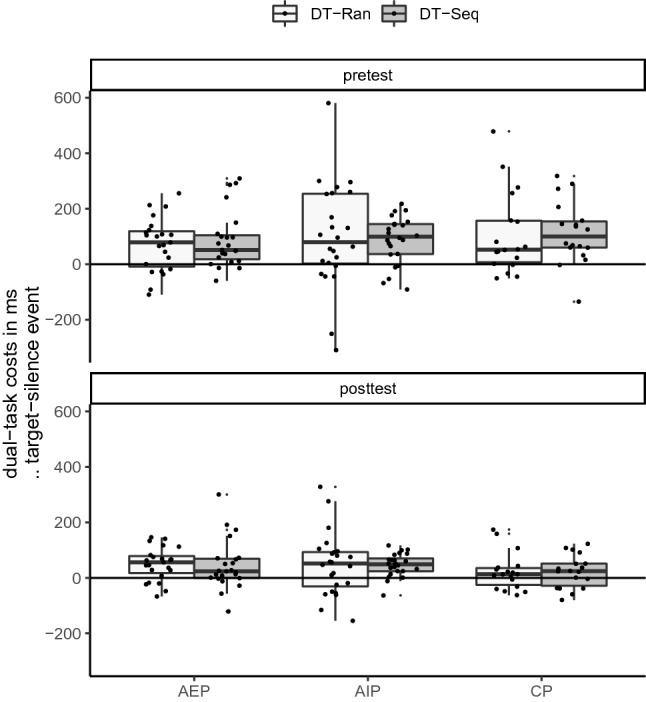
Boxplots of dual-task costs (Δ RT in ms: target—silence) depending on time (pretest, posttest) and sequence (sequence, random), separately for the practice groups (*AEP* action execution, *AIP* action imagery, *CP* control action)

To analyze the interaction between event and time, dual-tasks costs were calculated (Δ RT: target—silence). The distribution of dual-task costs is shown in Fig. 6. A mixed-model ANOVA with the between-factor practice group (AEP, AIP, CP) and the within-factors *time* (pretest, posttest), sequence (sequence, random), and event (target, distractor) was performed on dual-task costs. The significant main effect time, *F* (1, 64) = 21, *p* < 0.001, *η*^2^_p_ = 0.25, indicated significantly lower dual-task costs in the posttest (Δ *M* = 38 ms) than in the pretest (Δ *M* = 93 ms). This indicates that dual-task costs were reduced from pretest to posttest, independently from sequence blocks and practice groups which may indicate automatization of stimulus–response coupling (Giesen & Rothermund, 2015).

All other main effects and interactions were not significant (time x practice group: *F* (2, 64) = 1.7, *p* = 0.186, *η*^2^_p_ = 0.05; all others *η*^2^_p_ < 0.05).

### Sequence knowledge: free generation and recognition performance

**Fig. 7:**
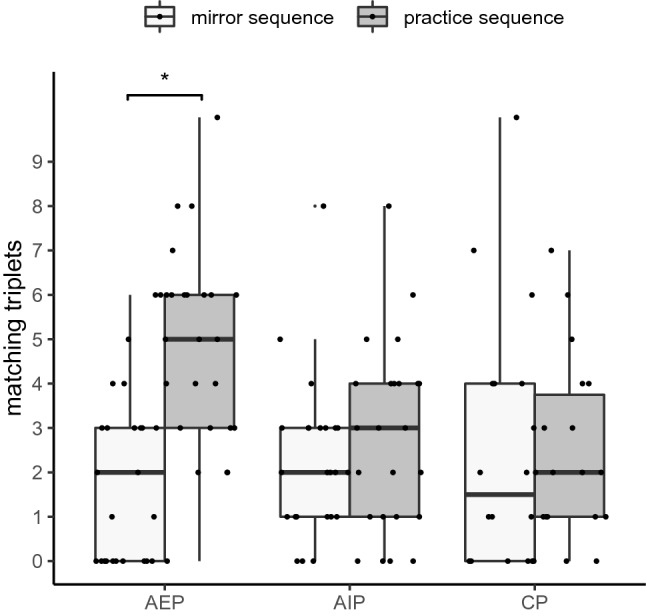
Boxplots of free generation performance of the practice sequence and its mirror sequence, separately for action execution practice (AEP), action imagery practice (AIP), and control practice (CP)

To measure explicit retrieval of the sequence, free generation performance was assessed as the number of triplets matching with the practice sequence and the number of triplets matching with the mirror sequence. A mixed-model ANOVA with the between-factor *practice group* (AEP, AIP, CP) and the within-factor *sequence* (practice, mirror) was performed on matching triplets. The distribution of the number of matching triplets is shown in Fig. 7.

The significant main effect *sequence*, *F* (1, 65) = 8.6, *p* = 0.005, *η*^2^_p_ = 0.12, was modified by the significant interaction between sequence and practice group, *F* (2, 65) = 5.3, *p* = 0.008, *η*^2^_p_ = 0.14. After AEP, the number of matching triplets was significantly higher with the practice sequence than with its mirror sequence (*p* < 0.001). Hence, participants of the AEP group were able to explicitly retrieve the practice sequence. After AIP and CP, the number of matching triplets did not significantly differ between the practice and mirror sequence (*p*_min_ = 0.403). The main effect *practice group* did not reveal significance, *F* (2, 65) = 2.8, *p* = 0.066, *η*^2^_p_ = 0.08.

**Fig. 8:**
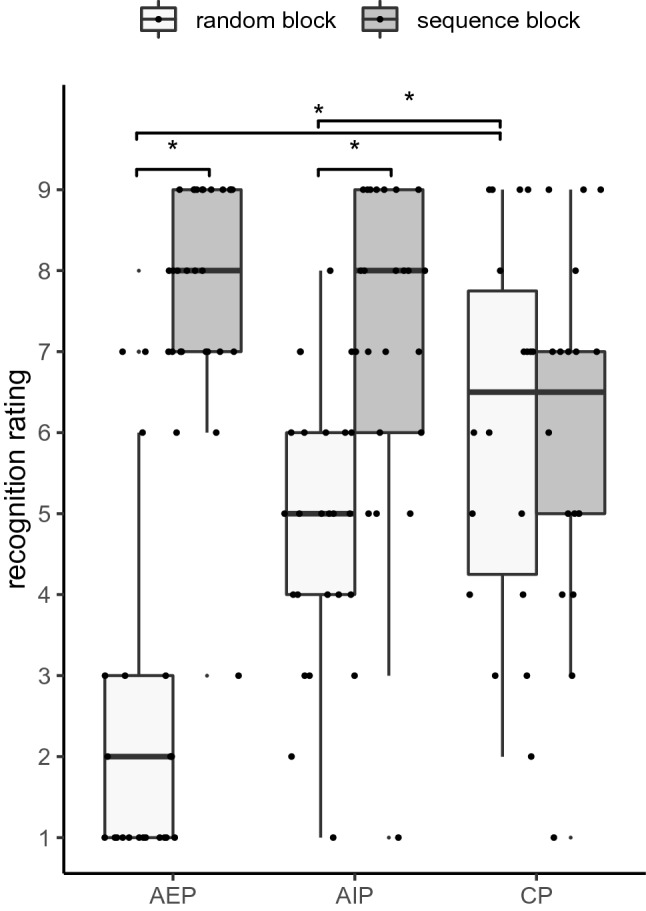
Boxplots of recognition ratings for a sequence block and a random block, separately for action execution practice (AEP), action imagery practice (AIP), and control practice (CP)

Recognition performance was assessed with participants’ self-ratings on how likely a performed sequence (sequence or random) coincided with the practice blocks. A mixed-model ANOVA with the between-factor *practice group* (AEP, AIP, CP) and the within-factor *sequence* (practice, random) was performed on recognition ratings. The distribution is shown in Fig. 8.

The significant main effect sequence, *F* (1, 65) = 43.6, *p* < 0.001, *η*^2^_p_ = 0.4, was modified by the significant interaction between sequence and practice, *F* (2, 65) = 16.3, *p* < 0.001, *η*^2^_p_ = 0.33. After AEP and AIP, the recognition ratings were significantly higher for the sequence block than for the random block (*p*_max_ = 0.001). Hence, participants in both groups were able to recognize the practice sequence. After CP, the recognition ratings did not significantly differ between the practice and random block (*p* = 0.938). Conversely, the difference between the sequences was significantly smaller in CP (Δ *M* = − 0.1) than in AIP (Δ *M* = 2.2; *p* = 0.049) and largest in AEP (Δ *M* = 5; *p*_max_ = 0.004). The main effect practice group did not reveal significance, *F* (2, 65) = 2, *p* = 0.145, *η*^2^_p_ = 0.06.

## Discussion

To investigate automatization in AIP, we compared dual-task and single-task performance in practice and random sequences after AIP, AEP, and CP. RTs decreased from pretest to posttest in both practice and random sequences in all groups indicating general sequence-unspecific learning. Further, RTs decreased to a greater extent in the practice sequence than in the random sequence after AIP and AEP, indicating sequence-specific learning. Dual-task costs were reduced independent from the performed sequence and independent from the practiced sequence structure indicating sequence-unspecific automatization. Further, participants were able to freely generate the sequence after AEP, but not after AIP and CP. Moreover, recognition of the sequence was stronger after AEP than after AIP, and not observable after CP.

### General sequence-unspecific learning

The analysis of RTs during practice indicated that sequence-unspecific learning occurred particularly in the first learning stages. RTs during practice in the CP group were reduced during the first practice sessions but remained quite stable from Session 6 onwards. Such general sequence-unspecific learning effects were further confirmed by the comparison of RTs in pretest and posttest which showed a significant reduction of RTs in all groups and all sequences. Such sequence-unspecific learning after AEP and AIP goes in line with previous findings in a serial reaction time task (Kraeutner et al., 2016). An improved maintenance of the home position on the keyboard as used during typing (Dahm & Rieger, 2019b; Long, 1976), may have led to improvements in all sequences and all groups. Alternatively, stimulus–response coupling occurred. That is, the mapping has been learned by intensifications of the association between stimuli and their corresponding responses (Schneider & Shiffrin, 1977).

### Sequence-specific learning

The analysis of RTs during practice indicated that sequence-specific learning occurred particularly in later stages of learning. RTs during practice became significantly shorter when the specific sequence was practiced (AEP) than in the control group that practiced random sequences (CP). Further, as expected, RTs in the posttest became significantly shorter in the practice sequence than in a random sequence after AEP and AIP (Dahm & Rieger, 2023; Dahm et al., 2022; Kraeutner et al., 2016; Solomon et al., 2021). In accordance with previous studies, such sequence-specific learning was significantly stronger in AEP than in AIP (Amemiya et al., 2010; Dahm & Rieger, 2023; Dahm et al., 2022; Ingram et al., 2016; Solomon et al., 2021). Beyond this replication, sequence-specific learning did not depend on cognitive load (single-task vs. dual-task) indicating that the acquired sequence-specific representation facilitates performance even under higher cognitive load conditions.

Unsurprisingly, significant differences between practice and random blocks were not observed after CP where random blocks were practiced. A previous study actually observed shorter RTs in sequence blocks than in random blocks after random practice (Frensch et al., 1999), but this may be caused by practice effects during the test blocks with a rather low-complex and discrete sequence of six elements in comparison to the eight-element sequence with random starting points in the present study.

### Dual-task costs and action automatization

Globally, we expected a reduction of dual-task costs as a consequence of motor automatization, as it is known to occur after extensive motor practice (Abernethy et al., 2012; Cohen & Poldrack, 2008; Fitts & Posner, 1967). Comparing single-task blocks and dual-task blocks, we failed to find motor automatization. Unexpectedly, significant dual-task costs were not observed in sequence blocks in the pretest, but in the posttest after AEP and AIP. Presumably, dual-task costs were not observed in the pretest because the main dual-task effect occurs only in one or two responses shortly after a tone event (see supplemental material). Tone events occurred only in 2.5–7.5% of all responses in dual-task blocks which decreased the chances to catch dual-task costs in the block-wise analysis. In contrast to this assumption, dual-task costs appeared, however, in the posttest. A reason for this could be that the decreased RT after practice boosted dual-task costs, as more elements are affected by a single dual-task event. Alternatively, using sequence representations after AEP and AIP (which were not available in the pretest), may have required attentional resources that increased dual-task costs (Curran & Keele, 1993).

For a deeper analysis, RTs after tone events (target, distractor, silence) were compared within dual-task blocks (Verwey et al., 2014) which revealed a reduction of dual-task costs. To be accurate here, it has to be mentioned that (matched) silence events during dual-task blocks are not real single-task events as the secondary task may still demand for resources, e.g., holding the number of counted tones in working memory (Verwey et al., 2014). However, in silence events, there is no simultaneous secondary task stimulus to be processed, which would demand for additional resources. Indeed, our data showed that dual-task processing mainly increased RTs of the first two responses following a tone (see supplementary material for analyses of the RTs following an event).

In the analysis on tone events, we observed a global sequence-unspecific reduction of dual-task costs, i.e., longer RTs after target and distractor tones than matched silence events. Because the reduction of dual-task costs from pretest to posttest was independent from the tested sequence structure (familiar vs. random) and the practiced sequence structure (sequence in AEP and AIP vs. random in CP), we assume that stimulus–response coupling (Giesen & Rothermund, 2015) rather than chunking of sequence elements (Immink et al., 2020) has been automatized; that does not necessarily mean that chunking of the sequence elements did not occur, but at least, it does not seem to contribute to a reduction of conscious cognitive processing. For discrete sequences, others found that a secondary task (i.e., tone counting) hampers familiar sequences less than unfamiliar sequences (Verwey et al., 2010). However, in line with the present study, using a continuous 12-element sequence, dual-task costs did not significantly differ between familiar and random sequences (Cohen & Poldrack, 2008). Thus, sequence-specific automaticity may evolve in discrete sequences, but not (or later) in continuous sequences. Further, the potential sequence-specific reduction in dual-task costs might be influenced by sequence complexity (e.g., number of elements) in continuous sequences. For instance, when using a continuous six-element sequence, dual-task costs were higher in familiar sequences than in random sequences (Curran & Keele, 1993).

Stimulus–response coupling has been proposed to be implicit (Giesen & Rothermund, 2015), but its automatization processes (via dual-task cost reductions) may not have been studied yet in AIP. One might argue that stimulus–response coupling reflects rather cognitive processes (Kraeutner et al., 2017) than motor performance to automatize. However, like in many actions in motor control, both, cognitive processes (e.g., stimulus processing and decision making) and motor processes (e.g., planning and executing motor responses) (Hommel, 1998), are involved in stimulus–response coupling of the implicit sequence learning paradigm. Further, the reduction of dual-task costs from pretest to posttest may be caused by a more efficient processing of the secondary task stimuli (e.g., speeding up of tone identification). However, the tones were not presented during 9 days of practice which makes this explanation unlikely. Moreover, analyses of the performance in the secondary task showed that counting errors did not significantly differ between pretest and posttest (see supplemental material). Indeed, in AEP, it has been shown that an explicit sequence learning setting results in automatization of abstract rules whereas an implicit sequence learning setting results in automatized stimulus–response coupling (Roeder & Ashby, 2016). Most importantly for the present study, such sequence-unspecific automatization occurred not only in AEP, but also in AIP. This is particularly interesting because we could show automatization of such in AIP. Hence, stimulus–response coupling reached a level of automaticity in AIP, although the action was not actually executed during practice. In accordance with this, automatic stimulus–response coupling has been found in the absence of actual action, when stimuli were presented simultaneously with verbal codes that denoted responses (Pfeuffer et al., 2017).

From a methodological perspective, it is interesting that the reduction of sequence-unspecific dual-task costs was not detectable in the comparison between single-task and dual-task blocks, but only in the comparison between tone and matched silence events within dual-task blocks. In the former, median RTs were not sensitive enough to detect dual-task costs, as they included mainly (unmatched) silence events (ca. 95%). Therefore, mean RTs of a complete dual-task block are considered less sensitive for dual-task costs than RTs shortly after events of the secondary task (Verwey et al., 2010).

### Awareness of the sequence

Results of the free generation and recognition test indicated the following: After AEP, participants were able to recall and recognize the practiced sequence, at least to a certain degree. After AIP, participants were not able to recall the practiced sequence, but to recognize it to a certain degree. Unsurprisingly, the CP group who practiced random sequences did not recall or recognize the sequence that has been practiced by the other groups.

Although sequence acquisition was implicit (no information about the sequence in advance), the acquired representation of the sequence allowed the AEP group to recall the sequence without stimulus presentation. We would like to emphasize the difference between (implicit/explicit) acquisition and (implicit/explicit) retrieval (Frings et al., 2020). Implicit learning (e.g., when explicit information is not provided in the instruction) may result in both, implicit and explicit retrieval abilities. However, this does not change the type of acquisition (implicit learning). If participants in the AEP group explicitly detected the sequence, this was not the result of external explicit instruction but rather the result of implicit inferences (by oneself) during practice. This was, however, not observed after AIP. According to this, AEP might evoke sequence representations that help to reproduce a sequence in the absence of external spatial stimuli, especially, when explicit knowledge is incomplete, and a rather implicit execution of the sequence was induced during practice. In contrast, being an explicit process itself (Glover & Baran, 2017), AIP evokes sequence representations in a spatial code (Dahm et al., 2022) that is rather explicit in nature and may require the presence of external stimuli. Alternatively, sequence reproduction and recognition may have been weaker in AIP than in AEP because sequence learning was generally weaker in AIP than in AEP as observed in the RTs.

One might be puzzled by the high values of recognition in the CP group which indicated that they highly recognized the sequence although they had practiced random blocks. The sequence structure they might have inferred from practice may have been that there were no response repetitions or that the sequence was random. The characteristic of randomness appeared familiar to them. Therefore, when performing short random and sequence blocks in the recognition task, they provided high recognition ratings. Although the absolute ratings of the recognition ratings in the control group may have little content validity, its relative meaning showed that ratings in the control group did not significantly differ between random and sequence blocks.

### Limitations and future directions

Results of the RTs during practice showed that imagination durations were significantly longer than execution durations. This stands in contrast to the assumption of functional equivalence (Jeannerod, 2001) and indicates that imagination and execution not only share some mechanisms (Dahm & Rieger, 2016a, 2016b; Jeannerod, 2001), but also differ considerably (Dahm & Rieger, 2019a, 2019b; Glover & Baran, 2017). An explanation for slower imagination durations than execution durations is that inhibitory mechanisms (Rieger et al., 2017) slow down the imagination process. Inhibition might be even more evident and demanding in the current experiment, as participants had to inhibit the motor execution of the sequence in parallel to a motor act of adjacent effectors (i.e., thumb pressing the space bar). Another explanation is that the explicit focus on the action is more detailed during imagination than the implicit focus on the action during execution (Glover & Baran, 2017). Further, perceptual information of different modalities that is processed in parallel during execution, may be processed in sequence during imagination (Glover & Baran, 2017). For instance, kinesthetic and visual information may be processed in parallel during execution, but in sequence in imagination, and thereby prolonging imagination durations.

One may argue that the participants did not imagine the actions during practice, but responded without paying attention to the screen and the response mapping, because the RTs were longer during AIP than during AEP and the imagination processes cannot be objectively controlled (Cumming & Eaves, 2018; Dahm, 2020). However, visual inspection of the RTs during practice of the analyzed participants showed a very similar learning curve in AIP and AEP, which indicates that they indeed practiced the sequences mentally by AIP. Most importantly, the sequence-specific learning effects after AIP strongly suggest that participants simulated the corresponding responses during AIP.

Further, it can be claimed that improvements of stimulus–response coupling in the AIP group may not be caused by imagery practice, but due to executing the task during the pretest. Hence, learning may not (only) have followed imagery practice, but the intermix of executed and imagined performance (Kraeutner et al., 2020). However, the AIP group had a break of 10 days without executed blocks between pretest and posttest and a ratio of 8/100 between executed and imagined blocks. Although this makes learning effects due to execution in the AIP group rather unlikely, we cannot rule out this argument for general sequence-unspecific learning effects and stimulus–response coupling. For sequence-specific effects, this does not hold. Both sequences were performed equally often during the pretest. Thus, differences in sequence acquisition were causally determined to different types of practice (AIP or AEP). To rule out such limitations for sequence-unspecific effects, future studies may, therefore, attempt to show sequence-specific automatization effects in AIP. In contrast to our assumptions about implicit sequence learning, we did not observe the expected reduction of sequence-specific dual-task costs. This was not even observed in AEP. Potentially, a discrete sequence production task with a fixed starting point may lead to an explicit sequence detection earlier and thereby to sequence-specific automatization effects rather than the implicit SRT used in the present study. Possibly, temporal characteristics of the task also constrain the detection of automatization effects (dual-task cost reductions). In contrast to previous studies observing sequence-specific automatization effects with tasks that were not limited in time (Gruetzmacher et al., 2011; Pfeifer et al., 2021; Verwey et al., 2015). In the present task, requiring participants to respond as fast as possible, led to shorter inter-keystroke intervals in sequence blocks than in random blocks, thereby reducing the time frame to process the secondary task particularly in sequence blocks. Further, participants could be informed before a test whether the subsequent sequence is random or fixed (Curran & Keele, 1993). This may reduce attentional demands during sequence execution. Future studies could investigate whether sequence-specific automatization effects can be observed after AIP in tasks where they appear in AEP to disentangle whether learning in AIP is cognitive, visual-motor, or motor in nature.

With pressing the space bar during practice, one might say that participants were practicing a dual-task which may have hampered sequence-specific automatization. However, sequence learning was observed as RTs were shorter in the practice sequence than in the random sequence. Further, pressing the space bar was equal in each response. It did not involve decisional processes or complex motor skills, which is why we believe that it was quickly integrated (Koch et al., 2018) into the serial reaction time task. But still, dissolving the two responses (target and space key) in the posttest may have hampered sequence-specific automatization effects. Moreover, the AIP demanded for inhibiting sequence-related responses without inhibiting the response of hitting the space bar, which at least can be described as a somehow artificial practice setting that might affect attentional capacities during performance and learning. Future studies may, therefore, integrate the additional keystrokes both in pretests, practice, and posttests to avoid problems of practice to test transfer or alternatively exclude additional keystrokes all over the experimental procedure to increase external validity (e.g., to have a pure AIP condition without any response production during practice).

We cannot foreclose to have missed a temporary increase of dual-task costs in early phases of learning, which possibly occurred using an implicit sequence learning approach (Chein & Schneider, 2012). As discussed earlier, the learners might have developed sequence knowledge that demands for attentional resources. Therefore, to detect sequence-specific automatization, future studies may add a third test for intermediate measurements of dual-task costs.

## Conclusion

The results showed sequence-specific learning, which was, however, not automatized in the present action, i.e., a sequence-specific reduction of dual-task costs was not observed. Thus, although a sequence representation was acquired, cognitive resources were not freed up during execution of the intensively practiced sequence when compared to random sequences. Instead of automatizing sequences of motor responses, participants may have improved the anticipation of the upcoming stimuli which also results in sequence learning (Koch & Hoffmann, 2000), but possibly not in a reduction of dual-task costs. We would assume that stimulus anticipation is a rather cognitive process in comparison to chunking of motor elements. This could also explain why sequence learning was observed in AIP which shows greater benefits for cognitive than for motor tasks (Driskell et al., 1994).

Further, we observed an automatization of sequence-unspecific learning indicated by a reduction of dual-task costs that was independent from the executed sequence and independent from the practiced sequence. We assume that stimulus–response coupling caused such general automaticity. Interestingly, automatization of the stimulus–response mapping was observed not only after AEP, but also after AIP. Hence, cognitive resources that are bound before practice, can be freed up after both AEP and AIP.

## Supplementary Information

Below is the link to the electronic supplementary material.

Supplementary file1 (PDF 409 KB)

## Data Availability

The author confirms that the data supporting the findings of this study are available within the article and its supplementary materials: https://osf.io/nuqx5/?view_only=f95ee1561c7b4b2991633dd7043f7c43. Determination of the sample size, all data exclusions, all manipulations, and all measures in the study are reported in the manuscript.
