# Supplementary material for: Imagine to automatize: automatization of stimulus–response coupling after action imagery practice in implicit sequence learning
Source: Psychol Res. 2023 Mar 4;87(7):2259–74. doi: 10.1007/s00426-023-01797-w (PMC10457413; doi:10.1007/s00426-023-01797-w)
Supplement: Supplementary file 1 — Supplementary file1 (PDF 409 KB) [file 426_2023_1797_MOESM1_ESM.pdf]

SUPPLEMENTAL MATERIAL

Imagine to automatize: Automatization of stimulus-response coupling after action imagery practice in implicit sequence learning

Stephan F. Dahm[1,2], Henri Hyna[3] & Daniel Krause[3]

[1] Universität Innsbruck, Department of Psychology, Innsbruck, Austria
[2] UMIT Tirol - Private University for Health Sciences and Health Technology, Hall in Tyrol, Austria
[3] Paderborn University, Department of Exercise and Health, Paderborn, Germany

Overview

This document includes additional analyses that may be of interest to some readers. In a first step, we report an analysis that includes the factor *position* which shows the effect of tone events on single reaction times of the serial reaction time task. In a second step, we analyzed visual, kinesthetic, and acoustic strength of representation during AEP, AIP, and CP. In a third step, we analyzed the responses in the tone counting task as a control calculation. In a fourth step, we analyzed the error rates.

1. Reaction time after tone events by position

To investigate the specific dual-task effects on reaction times, we calculated an ANOVA with the between-factor *practice group* (AIP: action imagery practice, AEP: action execution practice, CP: control practice) and the within-factors *time* (pretest, posttest), *sequence block* (sequence, random), *response position* (0, 1, 2, 3, 4), and *tone events* (target, distractor, silence). Means and standard errors of the reaction times are shown in Figure SM1. Results of the ANOVA are shown in Table SM1. Here, we focus on the main effect and interactions with the factor *position* which is not included in the manuscript. Tone events were target tones, distractor tones, or matched silence (no tones). The factor *response position* indicates whether the RTs stemmed from reactions directly after a tone event (1), from the second reaction after an event (2) or later reactions (3, 4, and 5).

The significant main effect *position* was modified by the significant *position* x time interaction, the significant *position* x *event interaction*, and the significant *position* x *time* x *event* interaction.

In short, RTs were significantly longer after target and distractor tones than after silence. As indicated by follow-up t-tests, these dual-task costs were significant only in position 0 ($p_{max} < .001$), in position 1 ($p_{max} < .001$), and position 2 ($p_{max} = .004$), but not in position 3 ($p_{min} = .116$) and position 4 ($p_{min} = .309$). Visual inspection of the data (see Figure SM1) further indicated that the occurrence of dual-task costs dependent on response speed. Because dual-task costs were primarily observed shortly after a tone event, we decided to average the data over the five responses after a tone event to simplify the analyses in the main manuscript.

Further, target and distractor tones differed significantly in the first positions in the posttest, but not in the pretest. At position 0, RTs were significantly longer after distractor tones than after target tones ($p = .037$). At position 1, this was reversed indicating significantly longer RTs after target tones than after distractor tones ($p = .029$). Ignoring the distractor tones of the secondary task immediately after their occurrence may have caused global inhibition (Rieger et al., 2017) which also prolonged reactions on the primary task. The longer RTs for target tones were most likely caused by working memory demands (i.e., memorization of the last number and adding one more; Frensch et al., 1999).

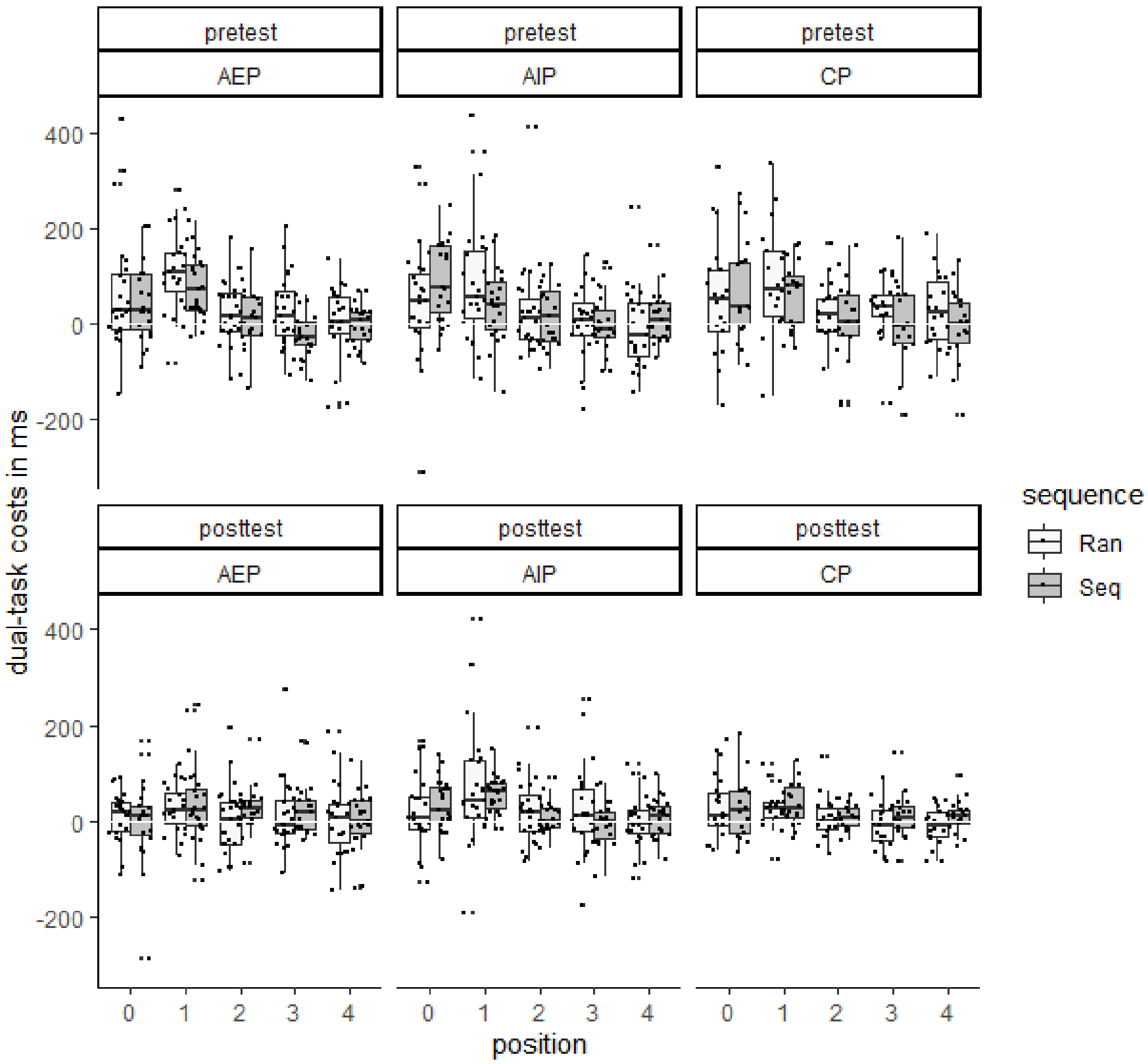

Figure SM1. Boxplots of reaction times (RT in ms) depending on time (pretest, posttest), practice group (AEP: action execution practice, AIP: action imagery practice, CP: control practice), response position (0, 1, 2, 3, 4), and tone event (target, distractor, silence) in A) sequence dual-task blocks and B) random dual-task blocks.

Table SM1. Statistical values of the ANOVA on RTs. Factors and factor levels were practice group (action imagery practice, action execution practice, control practice), time (pretest, posttest), sequence block (sequence, random), tone event (target, distractor, silence), and position (0, 1, 2, 3, 4). Only the effects and interactions with position are shown.

	F	*df1, df2*	*p*	η^2_p
position	59.6	3.3, 207.3	<.001	.49
position x practice group	1.6	6.7, 207.3	.141	.05
position x time	4.4	3.4, 213.6	.003	.07
position x sequence	0.6	3.9, 244.3	.657	.01
position x event	16.8	6.5, 402.5	<.001	.21
position x practice group x time	1.2	6.9, 213.6	.330	.04
position x practice group x sequence	0.5	7.9, 244.3	.877	.02
position x practice group x event	1	13, 402.5	.416	.03
position x time x sequence	0.7	3.5, 214.5	.602	.01
position x time x event	3.7	7.1, 439.8	.001	.06
position x sequence x event	1.6	7.3, 455	.120	.03
position x practice group x time x sequence	1.3	8, 16	.276	.02
position x practice group x time x event	0.9	14.2, 439.8	.533	.03
position x practice group x sequence x event	1	14.7, 455	.482	.03
position x time x sequence x event	1.2	8, 496	.276	.02
position x practice gr. x time x sequence x event	0.3	16, 496	.996	.01

2. Strength of representation during practice

To explore whether participants focused on the same content in AEP and AIP, we assessed the strength of kinesthetic, visual, and auditory representation after the last practice block in Session 10. Participants indicated on a rating scale (from 1 – 'not at all' to 9 – 'very strongly') how they felt/imagined to feel or saw/imagined to see or heard/imagined to hear how their fingers touched the keys. Means and standard errors of reported kinesthetic, visual, and auditory representations are shown in Figure SM2. An ANOVA with the between factor *practice group* (AEP, AIP, CP) and the within factor *modality* (kinesthesis, vision, acoustic) was conducted.

The significant main effect of *modality*, F (2, 130) = 114.2, $p < .001$, $\eta^2_p = .64$, indicated significantly stronger kinesthetic and acoustic representations than visual representations ($p_{max} < .001$), whereas kinesthetic and acoustic representations did not significantly differ ($p = .432$). Further, the significant main effect *practice group*, F (2, 65) = 9, $p < .001$, $\eta^2_p = .22$, was modified by the significant *practice group* x *modality* interaction, F (4, 130) = 11.4, $p < .001$, $\eta^2_p = .26$. Significantly stronger visual representations were reported after AIP than after AEP and CP ($p_{max} < .001$). Moreover, significantly stronger acoustic representations were reported after CP than after AEP ($p = .019$).

In short, the reported strength of representation of the AEP group indicates that the task required a stronger focus on kinesthetic and acoustic elements than on vision. However, in AIP visual elements were more important than in AEP and CP. Possibly, visual aspects of a movement play a key role in imagination, but not in execution of the present task. It remains unresolved why the CP group reported stronger acoustic representations than the AEP group.

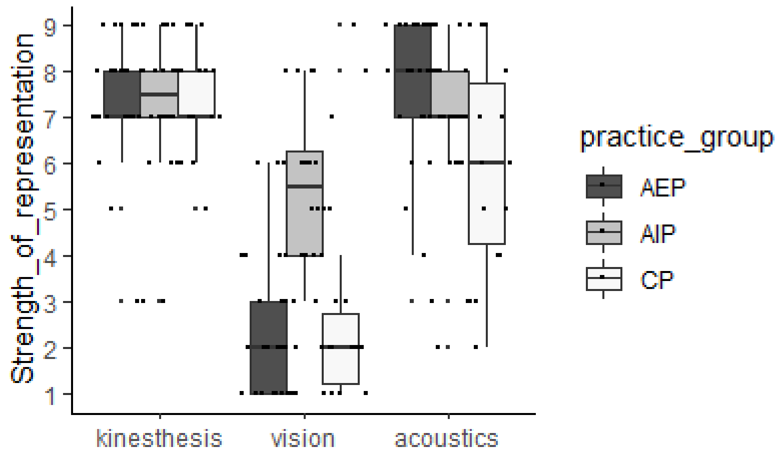

Figure SM2. Boxplots of reported kinesthetic, visual, and auditory strength of representation of the key presses during action-imagery practice (AIP), action-execution practice (AEP), and control practice (CP).

3. Counting errors

In a first step, counting errors during dual-task conditions were defined as the absolute difference between the number of reported target tones and actual target tones. Means and standard errors of the tone counting errors are shown in Figure SM3. A mixed ANOVA with the between-factor *practice group* (AIP, AEP, CP) and the within-factors *time* (pretest, posttest), and *sequence block* (sequence, random) was performed on TCT performance.

The significant main effect for *sequence block*, F (1, 65) = 7.8, p = .007, η^2_p = .11, indicated significantly fewer counting errors in sequence blocks (M = 0.16) than in random blocks (M = 0.3). The remaining effects were not significant: *practice group*: F (2, 65) = 1.1, p = .335, η^2_p = .03; *practice group* x *time*: F (2, 65) = 1.4, p = .261, η^2_p = .04; *practice group* x *sequence*: F (2, 65) = 3, p = .058, η^2_p = .08; all other F < 1.

In short, fewer counting errors occured in sequence blocks than in random blocks. This was however already observed in the pretest before the sequence was practiced.

Figure SM3. Mirrored density plots of the distribution of counting errors in the Tone Counting Task (TCT) in single-task blocks (ST-TCT), dual-task sequence blocks (DT-SEQ), and dual-task random blocks (DT-RAN) in the pretest and posttest separetely for the action-imagery practice group (AIP), the action-execution practice group (AEP), and the control practice group (CP). Note that the mean in the sequence block in the posttest of the AEP group was zero.

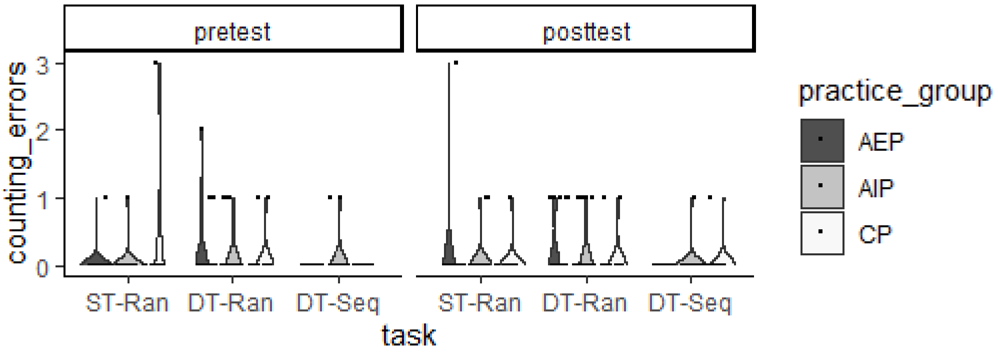

In a second step, dual-task costs in counting errors were calculated with the difference between counting errors in dual-task conditions and counting errors in single-task conditions of the tone counting task. Means and standard errors of dual-task costs on counting errors are shown in Figure SM4. A mixed ANOVA with the between-factor *practice group* (AIP, AEP, CP) and the within-factors *time* (pretest, posttest), and *sequence block* (sequence, random) was performed on dual-task costs in counting errors.

The significant main effect for *sequence block*, F (1, 65) = 7.8, p = .007, η^2_p = .11, indicated significantly fewer dual-task costs in sequence blocks (M = 0.08) than in random blocks (M = 0.22). The remaining effects were not significant: *practice group*: F (2, 65) = 1.1, p = .342, η^2_p = .03; *practice group* x *time*: F (2, 65) = 1.4, p = .261, η^2_p = .04; *practice group* x *sequence*: F (2, 65) = 3, p = .058, η^2_p = .08; all other F < 1.

In short, dual-task costs in counting errors were lower in sequence blocks than in random blocks. This was however already observed in the pretest before the sequence was practiced.

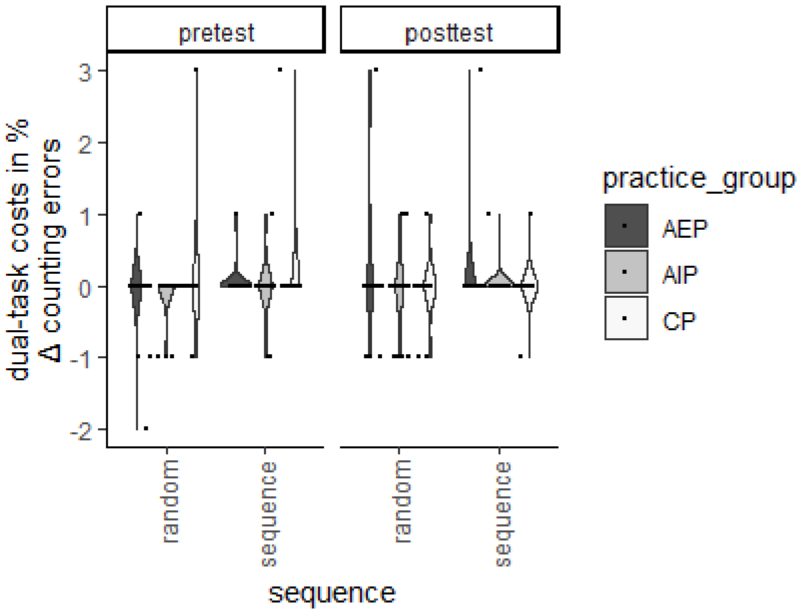

Figure SM4. Mirrored density plots of the distribution of dual-task costs in counting errors in the Tone Counting Task (TCT) in random and sequence blocks in the pretest and posttest separetely for the action-imagery practice group (AIP), the action-execution practice group (AEP), and the control practice group (CP). Note that the mean in the sequence block in the posttest of the AEP group was zero.

4. Error rates

Error rates were calculated as the percentage of incorrect responses during a block. An ANOVA with the between-factor *practice group* (AIP: action imagery practice, AEP: action execution practice, CP: control practice) and the within-factors *time* (pretest, posttest), *sequence block* (sequence, random), and *cognitive load* (single-task, dual-task) was performed on error rates. Means and standard errors of the error rates are shown in Figure SM5. Results of the ANOVA are shown in Table SM2.

The significant main effect *sequence block* indicated significantly more errors in random blocks (M = 6.3 %) than in sequence blocks (M = 3.9 %). The significant *practice group* x *time* x *sequence* interaction indicated that the difference between sequence blocks was increased from pretest to posttest in AEP ($p < .001$) and AIP ($p = .035$), but not in CP ($p = .514$). The significant main effect of *time* was modified by various interactions with *practice group*, *sequence*, and *cognitive load*. Significantly more errors in the posttest than in the pretest occurred in all conditions of the CP group ($p_{max} = .002$), in single-task random blocks and dual-task random blocks in AEP (p_{max} = .003), and in single-task random blocks in AIP ($p = .024$). The significant main effect *cognitive load* was modified by various interactions with *practice group*, *sequence*, and *time*. In the posttest, significantly fewer errors occurred in dual-task conditions than in single-task conditions in sequence blocks and random blocks in AEP (p_{max} = .001) and in sequence blocks in CP ($p = .038$).

In short, fewer errors were committed in sequence blocks than in random blocks already in the pretest. Maybe the repetitive eight-element structure of the sequence block made the blocks easier to perform. More importantly, after AEP and AIP the error rates increased in random blocks, but not in sequence blocks indicating sequence-specific learning. In the CP group who did not practice a specific sequence, the error rates increased in both, random and sequence blocks. This goes in line with the analysis of RTs in the main manuscript. Fewer errors occurred in dual-task conditions than in single-task conditions after AEP and CP. We speculate that higher error rates in single-task conditions resulted from interference due to explicit attention on the stimulus-response mapping. With high attentional load in dual-task conditions, the learners may have been forced to rely more on implicit and automatized processes, which might be more effective in this context. Interestingly, such a shift on attentional processes was not observed after AIP. Assuming that AIP relies more on explicit learning, participants may not have differed in sequence execution in single-task and dual-task blocks.

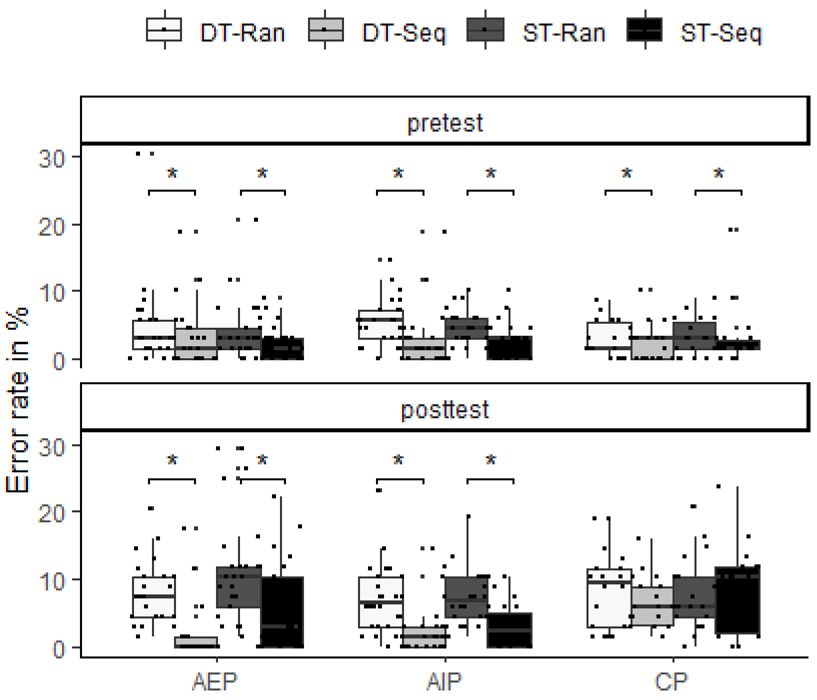

Figure SM5. Boxplots of the error rates (in %) depending on time (pretest, posttest), sequence block (Seq: sequence, Rand: random), and cognitive load (ST: single-task, DT: dual-task) separately for the practice groups (AEP: action-execution practice, AIP: action-imagery practice, CP: control practice).

Table SM2. Statistical values of the ANOVA on error rates. Factors and factor levels were practice group (action imagery practice, action execution practice, control practice), time (pretest, posttest), sequence block (sequence, random), and cognitive load (single-task, dual-task).

	F	*df1, df2*	*p*	η^2_p
practice group	0.4	2, 65	.652	.01
time	28.1	1, 65	<.001	.3
sequence	56.9	1, 65	<.001	.47
cognitive load	6.9	1, 65	.011	.1
practice group x time	4.4	2, 65	.016	.12
practice group x sequence	3.2	2, 65	.047	.09
practice group x load	3.4	2, 65	.038	.1
time x sequence	18.1	1, 65	<.001	.22
time x cognitive load	22.2	1, 65	<.001	.25
sequence x cognitive load	2.2	1, 65	.142	.03
practice group x time x sequence	3.9	2, 65	.026	.11
practice group x time x cognitive load	3.4	2, 65	.038	.1
practice group x sequence x load	<0.1	2, 65	.967	<.01
time x sequence x cognitive load	<0.1	1, 65	.909	<.01
practice group x time x sequence x cognitive load	0.8	2, 65	.463	.02

References

Frensch, P. A., Wenke, D., & Rünger, D. (1999). A secondary tone-counting task suppresses expression of knowledge in the serial reaction task. *Journal of Experimental Psychology: Learning, Memory, and Cognition*, *25*(1), 260–274. https://doi.org/10.1037/0278-7393.25.1.260

Rieger, M., Dahm, S. F., & Koch, I. (2017). Inhibition in motor imagery: A novel action mode switching paradigm. *Psychonomic Bulletin & Review*, *24*(2), 459–466. https://doi.org/10.3758/s13423-016-1095-5
